# Induction of Siglec-F^hi^CD101^hi^ eosinophils in the lungs following murine hookworm *Nippostrongylus brasiliensis* infection

**DOI:** 10.3389/fimmu.2023.1170807

**Published:** 2023-05-12

**Authors:** Alisha Chetty, Matthew G. Darby, Jamie Pillaye, A'ishah Taliep, Adam F. Cunningham, Matthew K. O’Shea, Gnatoulma Katawa, Laura E. Layland, Manuel Ritter, William G. C. Horsnell

**Affiliations:** ^1^ Wellcome Centre for Infectious Diseases Research in Africa, Institute of Infectious Disease and Molecular Medicine, Department of Pathology, Division of Immunology, University of Cape Town, Cape Town, South Africa; ^2^ Institute of Immunology and Immunotherapy, College of Medical and Dental Sciences, University of Birmingham, Birmingham, United Kingdom; ^3^ Unité de Recherche en Immunologie et Immunomodulation (UR2IM)/Laboratoire de Microbiologie et de Contrôle de Qualité des Denrées Alimentaires (LAMICODA), Ecole Supérieure des Techniques Biologiques et Alimentaires, Universite de Lomé, Lomé, Togo; ^4^ German Centre for Infection Research (DZIF), Neglected Tropical Disease, Partner site Bonn-Cologne, Bonn, Germany; ^5^ Institute for Medical Microbiology, Immunology and Parasitology (IMMIP), University Hospital Bonn (UKB), Bonn, Germany; ^6^ Laboratory of Molecular and Experimental Immunology and Neuro-genetics, Centre National de la Recherche Scientifique (CNRS)-University of Orleans and Le Studium Institute for Advanced Studies, Orléans, France; ^7^ Institute of Microbiology and Infection, University of Birmingham, Birmingham, United Kingdom

**Keywords:** helminths, *Nippostrongylus brasilienis*, eosinophils, Siglec-F, CD101, ILC2s, lung

## Abstract

Helminth-induced eosinophils accumulate around the parasite at the site of infection, or in parasite-damaged tissues well after the helminth has left the site. The role of helminth-elicited eosinophils in mediating parasite control is complex. While they may contribute to direct parasite-killing and tissue repair, their involvement in long-term immunopathogenesis is a concern. In allergic Siglec-F^hi^CD101^hi^, eosinophils are associated with pathology. Research has not shown if equivalent subpopulations of eosinophils are a feature of helminth infection. In this study, we demonstrate that lung migration of rodent hookworm *Nippostrongylus brasiliensis* (*Nb*) results in a long-term expansion of distinct Siglec-F^hi^CD101^hi^ eosinophil subpopulations. *Nb*-elevated eosinophil populations in the bone marrow and circulation did not present this phenotype. Siglec-F^hi^CD101^hi^ lung eosinophils exhibited an activated morphology including nuclei hyper-segmentation and cytoplasm degranulation. Recruitment of ST2^+^ ILC2s and not CD4^+^ T cells to the lungs was associated with the expansion of Siglec-F^hi^CD101^hi^ eosinophils. This data identifies a morphologically distinct and persistent subset of Siglec-F^hi^CD101^hi^ lung eosinophils induced following *Nb* infection. These eosinophils may contribute to long-term pathology following helminth infection.

## Introduction

Eosinophils are a canonical feature of helminth infection. Several helminths have life cycles that involve larval migration through the human lung tissue which can be associated with significant pathology such as Loeffler’s syndrome (1). However, our understanding of how lung eosinophil responses and, in particular, their heterogeneity are influenced by helminth infection is not as comprehensive as in the case of, for example, allergic inflammation (2–4).

Studies of allergic asthma have shown distinct roles of eosinophil subpopulations. For example, Siglec-F^hi^ ‘inflammatory’ eosinophil subsets in the allergic lungs are recruited after an allergen challenge and contribute to lung pathology (2, 5, 6). Conversely, Siglec-F_int_ ‘steady-state’ eosinophils associate with tissue homeostasis and protection from pathology (2, 4). The induction of such eosinophil subpopulations following helminth infection is incompletely understood.

Lung involvement is a feature of the life cycle of many common human helminth infections (e.g., *Ascaris* sp. and hookworm), and subsequent pathology may result from parasite migration through the lungs prior to the establishment of the patent intestinal infection (7, 8). Such pathology can be striking; for example, infection with murine hookworm *Nippostrongylus brasiliensis* (*Nb*) can result in significant mechanical damage to the alveolar architecture following the larval transition from the circulatory system into the airways (9). This leads to a significant accumulation of eosinophils in the lungs and the presentation of acute allergic-like pathology (10–12). While this acute damage is transient, mice typically develop a chronic emphysema-like pathology (13). The relevance of this modeling to human pathology is supported by a recent study identifying human exposure to *Ascaris lumbricoides* associated with a decline in lung function later in life (14).

In this study, we investigated the influence of *Nb* infection on the composition of Siglec-F^hi^ and Siglec-F_int_ eosinophils in the murine lungs. We also studied the longevity of these expanded populations in the lungs following parasite clearance and the contribution of *Nb*-elicited ILC2s in driving the accumulation of eosinophil subpopulations. We report that *Nb* infection leads to the accumulation of a predominant and persistent Siglec-F^hi^ eosinophil subset in the lungs and the expansion of this population requires the presence of non-T cell lymphocytes and is associated strongly with recruited ILC2 populations.

## Materials and methods

### Ethics statement

This study was carried out in accordance with the South African Bureau of Standards guidelines for animal work and approved by the UCT Faculty of Health Sciences Animal Ethics Committee (Project licenses 018-041, 021-012). All researchers were accredited by the South African Veterinary Council.

### Animals

Wild-type BALB/c, athymic *Nude*, and IL-4Rα^-/-^ (BALB/c background) mice aged 6 to 8 weeks were obtained from the specific pathogen-free breeding facility and housed in the BSL2 experimental unit at the Faculty of Health Sciences Research Animal Facility, University of Cape Town, South Africa.

### *N. brasiliensis* infection

*Nb* was maintained in Wistar rats as previously described (15). Briefly, rats were infected subcutaneously with 5000x infectious *Nb* L3 larvae. Feces were collected at day 6 to 8 post-infection and cultured for ~1 week to obtain hatched L3. Mice were subcutaneously infected with 500x L3 delivered in sterile water. For secondary/recall infections, mice were infected 5 weeks after the initial *Nb* infection.

### FTY720 treatment

To block lymphocyte egress from secondary lymph tissues, mice received daily intraperitoneal treatment with 0.5 mg kg^-1^ FTY720 (Sigma-Aldrich) as previously described (16).

### Histology and immunohistochemistry

Isolated lung tissue was immediately fixed in a 10% phosphate-buffered formalin solution (Sigma-Aldrich), embedded, and sectioned (Leica Biosystems). Sections were stained with Sirius red and hematoxylin. To detect eosinophil cationic protein (ECP), antigen retrieval was performed in Citrate buffer (pH 6). Sections were blocked (5% BSA in PBS) and incubated overnight at 4°C with rabbit polyclonal anti-mouse RNASE3/ECP (abcam PA5-79927). Immunohistochemical detection of ECP was performed using EnVision Detection Systems (DAKO). Sections were viewed with an Axioskop Microscope (Zeiss) and images were taken with an AxioCam HRc and AxioVision 4.7 supporting software (Zeiss).

### Preparation of single cells

Bone marrow-derived cells were obtained from individual mice by flushing the right femur with sterile PBS. Cells were washed with PBS and resuspended in a staining buffer (PBS + 0.5% BSA and 2mM Ethylenediaminetetraacetic acid (EDTA)) for flow cytometry staining. Whole blood was obtained from individual mice by cardiac puncture (post-mortem) in 10% vol/vol 0.5M EDTA. Red blood cells were lysed in ACK lysis buffer (Gibco™ Thermo Fisher). Cells were washed with sterile PBS and resuspended in a staining buffer until flow cytometry staining. Whole lung was isolated from individual mice, minced, and digested in Dulbecco’s Modified Eagle Medium (Gibco™ Thermo Fisher) supplemented with 100 U/ml penicillin, 100mg/ml streptomycin, 25 μg/ml Liberase™ TL (Roche), and 13 μg/ml DNase (Roche) for 1 hour at 37°C with gentle shaking. Digested lung tissue was passed through a 70 μm cell strainer (Corning^®^ Sigma-Aldrich) and washed in sterile phosphate-buffered saline (PBS; Gibco™ Thermo Fisher). Red blood cells were lysed in ACK lysis buffer. Cells were washed and resuspended in a staining buffer.

### Flow cytometry

Cells were stained in a staining buffer containing 2% heat-inactivated rat sera, 1 μg anti-mouse CD16/32 (clone: 93, BioLegend), and fluorochrome-conjugated antibodies in the dark for 20 minutes at 4°C. Eosinophils were identified using CD45 Alexa Fluor^®^ 700 (clone: 30-F11, BioLegend), CD11b Brilliant violet (BV) 421™ (clone: M1/70, BioLegend), Ly6C FITC (clone: HK1.4, BioLegend), Ly6G APC Cy7 (clone: 1A8, BioLegend), and Siglec-F PE (clone: S17007L, BioLegend). ILC2s were characterised using CD45 Alexa Fluor^®^ 700 (clone: 30-F11, BioLegend), lineage cocktail PE (CD3ϵ clone: 145-2C11, Ly-6G/Ly-6C clone: RB6-8C5, CD11b clone: M1/70, CD45R/B220 clone: RA3-6B2, TER-119 clone: Ter-119, BioLegend), IL-7Rα (CD127) PE Cy7 (clone: A7R34, BioLegend), ICOS APC (clone: C398.4A, BioLegend), ST2 (IL-33Rα) BV421™ (clone: DIH9, BioLegend), and KLRG1 BV510™ (clone: 2F1/KLRG1, BioLegend). 7-aminoactinomycin D (7-AAD) staining was used to identify non-necrotic (‘Live’) cells. Cells were acquired on an LSRFortessa (BD Biosciences) and FACS Aria I (BD Biosciences) for cell sorting. Single-stained and unstained controls were used to compensate for spectral overlap. Data were analyzed using FlowJo^©^ V10 (Treestar, Ashland, OR).

### Cytospins and microscopy imaging

FACS-sorted eosinophil subpopulations were centrifuged at 800rpm for 10 min in cytospin chambers (Shandon Cytospin 4, Thermo Fisher) and transferred onto glass slides (Sigma-Aldrich). Slides were air-dried, fixed in ice-cold methanol, and stained using Wright-Giemsa stain (Sigma-Aldrich) to visualize the cell nucleus and cytoplasm. Cytospins were imaged using a Zeiss Axioskop Microscope (Zeiss) and AxioCam HRc and AxioVision 4.7 supporting software.

### Statistics

Statistical analysis was performed using Prism 6 (GraphPad). Data is represented as mean ± s.e.m. The relevant details on the statistical tests performed can be found in the figure legends. A P value < 0.05 was considered significant.

## Results

### *Nb* infection induces Siglec-F^hi^CD101^hi^ eosinophils in the lungs

**Figure 1:**
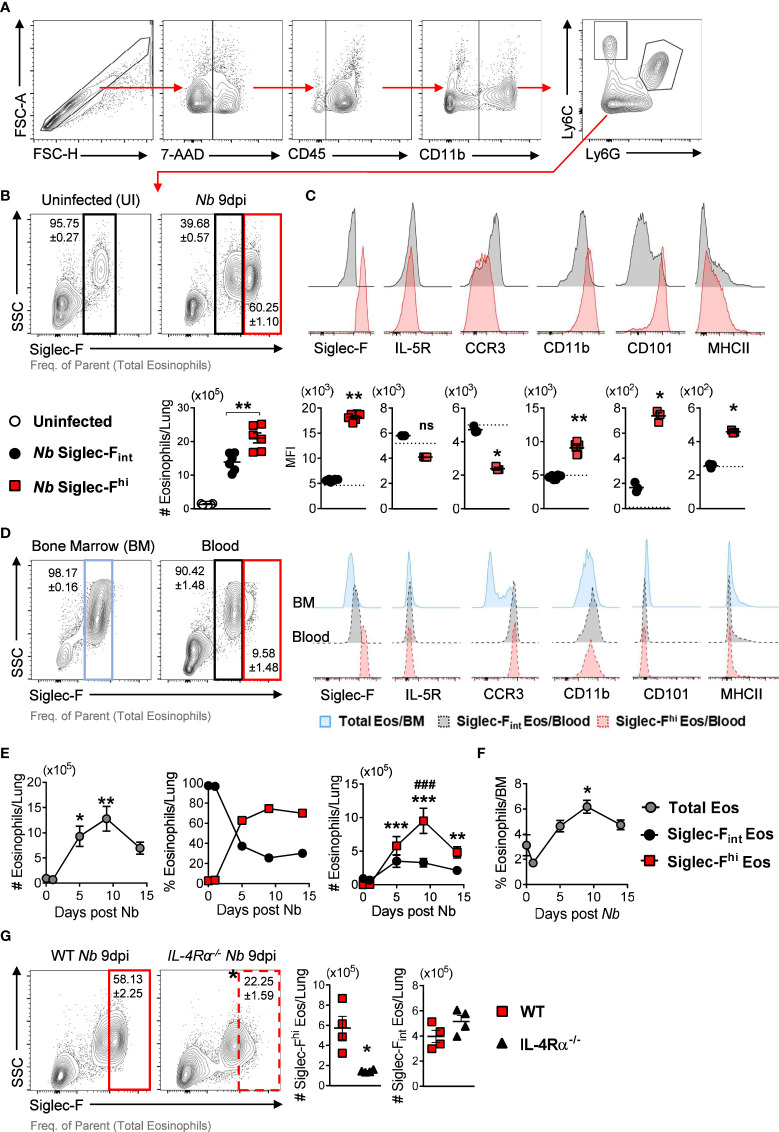
*Nb* infection induces lung eosinophil populations with differential expression of Siglec-F after parasite clearance: **(A)** Gating strategy to identify eosinophils by flow cytometry. **(B)** Proportions and numbers of Siglec-F^hi^ and Siglec-F_int_ subpopulations in the lungs at day 9 post-*Nb* infection (*Nb* 9dpi), compared to uninfected mice. **(C)** Representative histograms and MFI of Siglec-F, IL-5R, CCR3, CD11b, CD101, and MHCII expression on lung Siglec-F^hi^ (red) and Siglec-F_int_ (black) eosinophils. The dotted lines represent the MFI of lung eosinophils from uninfected mice. **(D)** Eosinophil proportions and phenotypes in the bone marrow and blood of *Nb*-infected mice at day 9 post-infection. **(E)** Total Siglec-F^hi^ and Siglec-F_int_ lung eosinophils and **(F)** proportions of total bone marrow (BM) eosinophils at day 0 (uninfected), 1, 5, 9, and 14 post-*Nb* infections. **(G)** Proportions and numbers of Siglec-f^hi^ lung eosinophils in *Nb*-infected WT and IL-4Rα global knock-out (*IL-4Rα^-/^* ^-^) mice at *Nb* 9 dpi. Data is representative of two independent experiments (4 to 6 mice per group). Statistical analysis was performed using a Mann Whitney t test, a Kruskal-Wallis ANOVA with Dunn’s multiple comparisons tests, or two-way ANOVA with Bonferroni’s multiple comparisons tests. *p ≤ 0.05, **p ≤ 0.01, ***p ≤ 0.001, and ^###^ *p* ≤ 0.001. For **(E)**: * - compared to Uninfected; ^#^ - comparing Siglec-F^hi^, and Siglec-F_int_ eosinophils at each time point. Black arrows, Flow plot axis; Red arrows, Gating sequence. ns, not significant.

We characterized lung eosinophil responses following *Nb* infection (**Figure 1A**). Siglec-F_int_ steady-state lung eosinophils were predominant in uninfected mice. This eosinophil population increased following *Nb* infection (*Nb* 9dpi) but was surpassed in number and proportion by Siglec-F^hi^ eosinophils (**Figure 1B**). This Siglec-F^hi^ eosinophil expansion in the lungs following *Nb* infection was independent of gender (**Supplementary Figure 1A**). *Nb*-expanded Siglec-F_int_ lung eosinophils expressed similar levels of cell surface markers Siglec-F, IL-5R, CCR3, CD11b, CD101, and MHCII as steady-state eosinophils from uninfected mice (**Figure 1C**). *Nb*-induced Siglec-F^hi^ eosinophils expressed higher levels of CD101, CD11b, and MHCII and lower expression of CCR3, compared to Siglec-F_int_ eosinophils (**Figure 1C**).

Others have suggested that Siglec-F^hi^ eosinophils are terminally differentiated cells that are recruited to the lungs (2, 17). We did not find Siglec-F^hi^CD101^hi^ eosinophils in the bone marrow (**Figure 1D**). We observed a small population of Siglec-F^hi^ eosinophils in the circulation which did not express high levels of CD101 (**Figure 1D**). To mitigate blood contamination in the lung tissue, we performed PBS perfusion following routine exsanguination *via* cardiac puncture and found equivalent proportions and numbers of Siglec-F^hi^CD101^hi^ lung eosinophils with exsanguination + PBS-perfusion compared to exsanguination only (**Supplementary Figures 1B, C**). This suggests that following *Nb* infection, eosinophils recruited from the bone marrow may only acquire the full Siglec-F^hi^CD101^hi^ phenotype once they have migrated to the lungs. We did not observe Siglec-F^hi^CD101^hi^ eosinophils in the intestine-draining mesenteric lymph nodes (**Supplementary Figure 2**) following *Nb* infection.

Longitudinal analysis over the course of *Nb* infection (day 0, 1, 5, 9, and 14 PI) revealed that peak levels of total and Siglec-F^hi^ lung eosinophils occurred at day 9 post-infection, approximately 7 days after the parasite had left the lungs (**Figure 1E**). This time point also coincides with parasite clearance from the intestine and peak type 2 cytokine responses in the lungs (11, 18–20). The *Nb*-induced increase in lung eosinophils was also mirrored in the bone marrow (**Figure 1F**). While total eosinophil numbers started to decline over time in the lungs, proportionally Siglec-F^hi^ subsets continued to predominate (**Figure 1E**). Siglec-F^hi^ eosinophils in the lungs were significantly reduced in IL-4Rα knock-out mice (IL-4Rα^-/-^) when compared to wild-type mice, with no significant difference in Siglec-F_int_ eosinophil numbers between these groups (**Figure 1G**). This suggests type 2 immune signaling (i.e., IL-4 and IL-13) contributes to the expansion of Siglec-F^hi^ lung eosinophils following *Nb* infection.

### *Nb*-induced Siglec-F^hi^CD101^+^ lung eosinophils have a distinct morphology in comparison to steady-state and *Nb*-induced Siglec-F_int_CD101^-^ lung eosinophils

**Figure 2:**
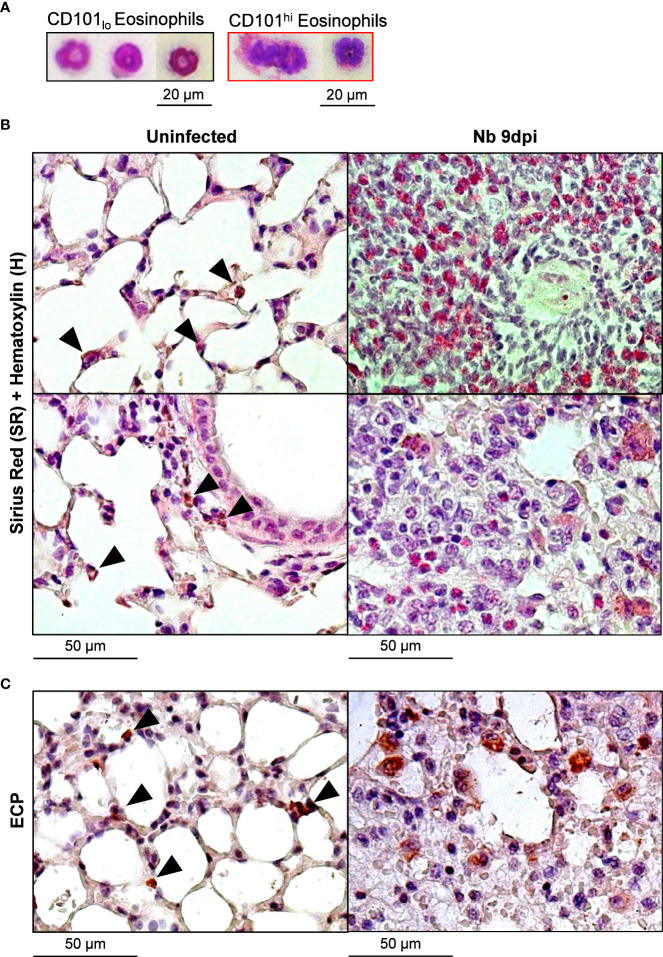
*Nb*-induced Siglec-F^hi^CD101^hi^ lung eosinophils have a unique morphology to steady-state and *Nb*-induced Siglec-F_int_CD101^-^ eosinophils: Lung eosinophil subpopulations at day 9 post-*Nb* infection *(Nb* 9dpi) were sorted by flow cytometry and visualized using modified Giemsa-Wright staining. **(A)** Representative images of CD101_lo_ and CD101^hi^ lung eosinophils (x400 magnification). Lung tissue was fixed and analyzed by immunohistochemistry. Representative images of **(B)** Sirius red (SR) and Hematoxylin **(H)**, and **(C)** eosinophil cationic protein (ECP)-stained sections were taken at x200 magnification. Black arrowheads identify eosinophils at a steady state. Data is representative of two independent experiments (3 to 6 mice per group).

We assessed the morphology of lung eosinophils following *Nb* infection and found CD101_lo_ eosinophils exhibited ring-shaped nuclei while CD101^hi^ eosinophils had hyper-segmented nuclei and less dense cytoplasm (**Figure 2A**), resembling steady-state and activated/inflammatory phenotypes, respectively (2, 3, 21). Further histological analysis of uninfected (steady state) and *Nb* 9dpi lung tissue showed recruited eosinophils displayed an activated morphology following *Nb* infection (**Figure 2B**). While both steady-state and *Nb*-induced eosinophils were positive for eosinophil cationic protein (ECP), *Nb*-induced eosinophil ECP was dispersed (**Figure 2C**), suggesting eosinophil degranulation in the tissue (22).

### *Nb*-induction of Siglec-f^hi^CD101^hi^ eosinophils is associated with ILC2 recruitment to the lungs independent of type 2 CD4^+^ T cell responses

**Figure 3:**
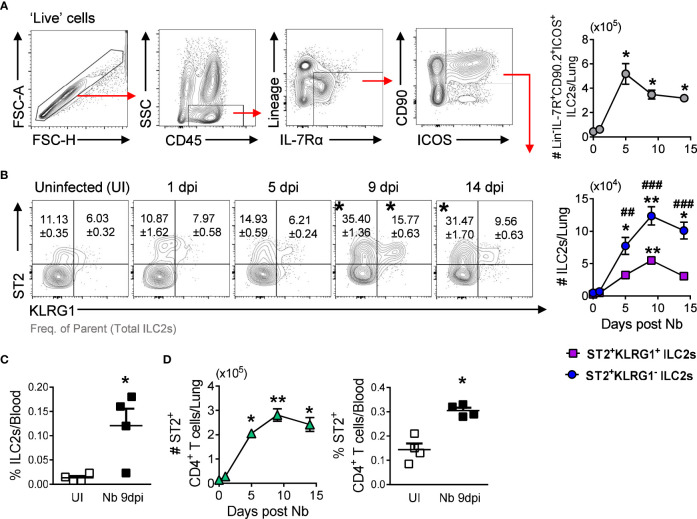
ST2^+^ CD4^+^ T cells and ILC2s peak levels in the lungs track with Siglec-F^hi^ eosinophil expansion: **(A)** ILC2 gating strategy and total lung ILC2s numbers at day 0 (Uninfected (UI)), 1, 5, 9, and 14 post-Nb infections. **(B)** ST2^+^KLRG1^-^ and ST2^+^KLRG1^+^ ILC2 proportions and numbers in the lungs. **(C)** Levels of circulating ST2^+^ ILC2s in uninfected and *Nb* 9dpi mice. **(D)** Numbers and proportions of ST2^+^ CD4^+^ T cells in the lungs and circulation. Data is representative of two independent experiments (4 to 6 mice per group). Statistical analysis was performed using a Mann Whitney t test, a Kruskal-Wallis ANOVA with Dunn’s multiple comparisons tests, or two-way ANOVA with Bonferroni’s multiple comparisons tests. *p ≤ 0.05, **p ≤ 0.01, ^#^ *p* ≤ 0.05, ^##^ *p* ≤ 0.01, and ^###^ *p* ≤ 0.001. For **(B)**: * - compared to Uninfected, ^#^ - comparing ST2^+^KLRG1^-^ and ST2^+^KLRG1^+^ ILC2s at each timepoint. Black arrows, Flow plot axis; Red arrows, Gating sequence.

Innate type 2 lymphoid cells (ILC2s) are essential for the induction of type 2 immunity and mediate lung eosinophil expansion by secreting the type 2 cytokines IL-5 and IL-13 (23, 24). As expected, we observed a significant increase in total lung ILC2s at day 5 post-*Nb* infection (**Figure 3A**), which paralleled the expansion of Siglec-F^hi^ eosinophils (**Figure 1E**). Previous studies have characterized IL-33-responsive tissue-resident ILC2s (nILC2s) and recruited IL-25-responsive inflammatory ILC2s (iILC2s) by differential expression of the IL-33 receptor (ST2) and killer cell lectin-like receptor subfamily G member 1 (KLRG1) (25–27). We observed a significant expansion of ST2^+^KLRG1^-^ ILC2s in the lungs following *Nb* infection, substantially more than ST2^+^KLRG1^+^ ILC2s (**Figure 3B**). We did not find ST2^lo^KLRG1^+^ iILC2s (25, 26); however, ILC2 phenotypes are influenced by mouse strain, with lung ILC2s from BALB/c mice being reported to express lower levels of KLRG1 compared to C57BL/6 mice (28). Additionally, we found raised levels of ILC2s in the blood at day 9 post-*Nb* infection (**Figure 3C**), supporting the expansion of lung ILC2s to be attributed to cell trafficking from another site, as demonstrated by others (27, 29, 30). We also observed an increase in ST2^+^ CD4^+^ T cells in the lungs and blood following *Nb* infection (**Figure 3D**).

**Figure 4:**
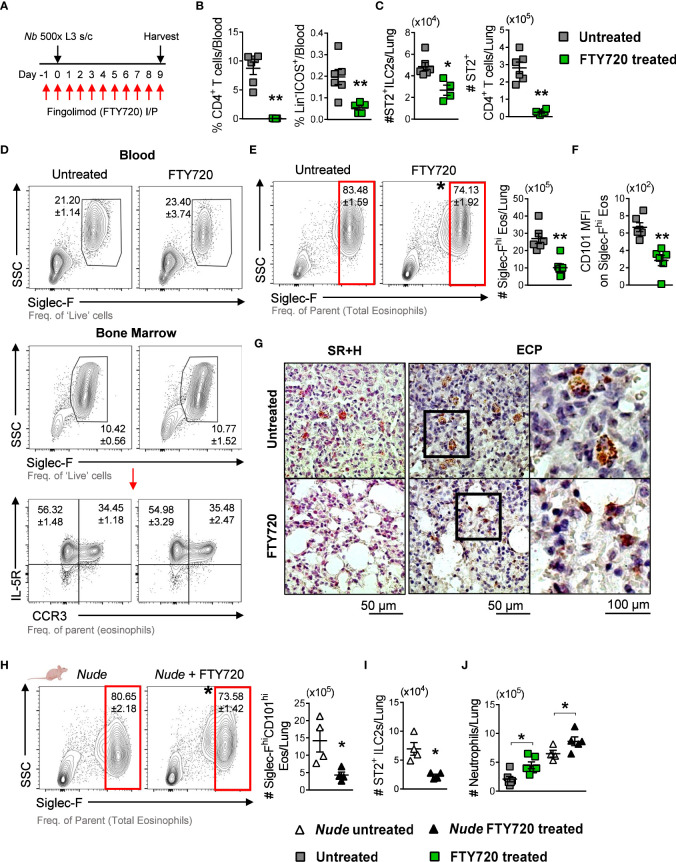
*Nb*-induced Siglec-F^hi^CD101^+^ lung eosinophils are associated with recruited ST2^+^ ILC2s: **(A)** Mice were treated with FTY720 from -1 until 9 dpi to block lymphoid migration to the lungs. **(B)** Circulating CD4^+^ T cells and Lin^-^ICOS^+^ cells in untreated and FTY720-treated mice, measured at *Nb* 9dpi by flow cytometry. **(C)** Numbers of ST2^+^ ILC2s and CD4^+^ T cells in the lungs at 9dpi. **(D)** Proportions of total blood eosinophils and total IL-5R^+^CCR3^-^ and IL-5R^+^CCR3^+^ bone marrow eosinophils in untreated and FTY720-treated *Nb* 9dpi mice. **(E)** Proportions and numbers of Siglec-F^hi^ eosinophils and **(F)** mean CD101 expression on Siglec-F^hi^ eosinophils in the lungs. **(G)** Representative images of Sirius red and Hematoxylin (SR+H) and eosinophil cationic protein (ECP)-stained lung sections were taken at x200 and x400 magnification. Athymic *Nude* mice were treated with FTY720 prior to and during *Nb* infection: **(H)** Proportions and numbers of Siglec-F^hi^CD101^hi^ eosinophils and **(I)** ST2+ ILC2s in the lungs of untreated and FTY720-treated *Nude* mice (*Nb* 9dpi). **(J)** The number of neutrophils in the lungs of WT and *Nude* untreated and FTY720-treated mice at *Nb* 9dpi. Data is representative of two independent experiments (4 to 6 mice per group). Statistical analysis was performed using a Mann Whitney t test. *p ≤ 0.05 and **p ≤ 0.01. Black arrows, Flow plot axis; Red arrows, Gating sequence; #, Cell numbers.

We then tested whether ST2^+^ lymphoid recruitment to the lungs mediated induction of Siglec-F^hi^ lung eosinophils by treating *Nb*-infected mice with Fingolimod (FTY720) to block the trafficking of lymphoid cells to the lungs (**Figure 4A**) (27, 31). FTY720 treatment resulted in a significant reduction in CD4^+^ T cells and Lin^-^ICOS^+^ cells in circulation, compared to untreated mice (**Figure 4B**). This impaired lymphoid trafficking resulted in a significant reduction in ST2^+^ ILC2s and ST2^+^ CD4^+^ T cells in the lungs of FTY720-treated *Nb* 9dpi mice, compared to untreated counterparts (**Figure 4C**). Sugita et al., 2010 reported that FTY720 treatment can alter the egress of eosinophils from the bone marrow (32). We found that eosinophil development in the bone marrow and egress into circulation was not impacted by FTY720 treatment (**Figure 4D**). However, impaired lymphoid recruitment resulted in a significant reduction in the proportion and number of Siglec-F^hi^ eosinophils in the lungs (**Figure 4E**). Further analysis revealed that CD101 expression was also significantly lower on Siglec-F^hi^ lung eosinophils in FTY720-treated mice, compared to untreated mice (**Figure 4F**). The proportions and phenotypes of Siglec-F_int_ lung eosinophils were equivalent in FTY720-treated and untreated mice, while total and Siglec-F_int_ eosinophil cell numbers were reduced in the lungs of FTY720-treated mice at *Nb* 9dpi (**Supplementary Figure 3**). Histological examination of the lung tissue revealed that lung eosinophils in FTY720-treated mice did not exhibit activated characteristics as in untreated *Nb*-infected mice (**Figure 4G**). These data support recruited lymphoid cells mediating accumulation of Siglec-F^hi^CD101^hi^ activated eosinophils in the lungs following *Nb* infection.

Lung-resident CD4^+^ T cells are also important contributors to immunity against *Nb* infection (16). To test whether lung CD4^+^ T cells contributed to the accumulation of Siglec-F^hi^ eosinophils, we treated athymic *Nude* mice (characterized by impaired CD4^+^ T cell responses) with FTY720 following *Nb* infection. Proportions of Siglec-F^hi^ lung eosinophils were equivalent in WT and *Nude Nb* 9dpi mice (WT 83.48 ± 1.59% *vs*. *Nude* 80.65 ± 2.18%); however, FTY720-treated *Nude* mice had significantly lower proportions and numbers of Siglec-F^hi^CD101^hi^ lung eosinophils, compared to untreated *Nude* mice (**Figure 4H**). This was accompanied by a significant reduction in ST2^+^ ILC2s in the lungs of FTY720-treated *Nude* mice, compared to untreated counterparts (**Figure 4I**). These data support the findings that *Nb*-induced Siglec-F^hi^CD101^hi^ lung eosinophil accumulation requires the recruitment of ST2^+^ ILC2s and is independent of TH2 CD4^+^ T cell responses.

With a reduction in Siglec-F^hi^CD101^hi^ eosinophils in the lungs, we also observed significantly increased numbers of lung neutrophils in FTY720-treated *Nb*-infected mice, compared to uninfected controls (**Figure 4J**).

### *Nb*-induced Siglec-F^hi^CD101^hi^ lung eosinophils are maintained long after the infection has cleared

**Figure 5:**
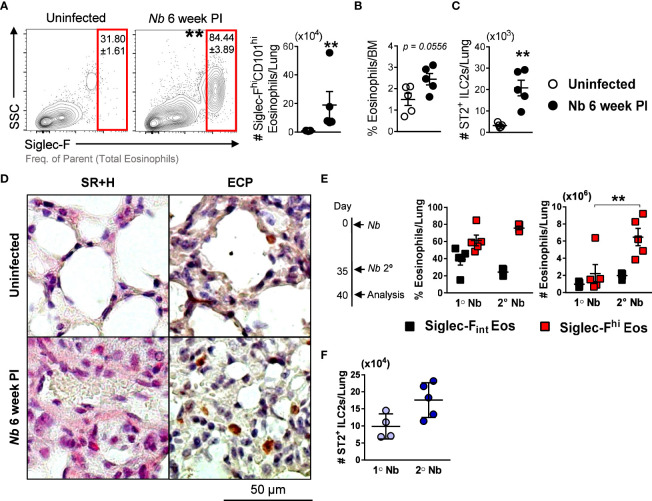
Numbers of *Nb*-induced Siglec-F^hi^CD101^hi^ lung eosinophils are maintained after parasite clearance and amplified after secondary infection: **(A)** Proportions and numbers of Siglec-F^hi^CD101^hi^ inflammatory eosinophils in the lungs at 6 weeks post *Nb* infection, compared to uninfected mice. **(B)** Proportions of total eosinophils in the bone marrow (BM) and **(C)** numbers of ST2^+^ ILC2s in the lungs. Lung tissue was fixed and analyzed by immunohistochemistry. **(D)** Representative images of Sirius red and Hematoxylin (SR+H) and eosinophil cationic protein (ECP)-stained sections were taken at x200 magnification. To analyze lung eosinophils responses during a secondary/recall infection, mice were infected with *Nb 5* weeks following primary infection. **(E)** Proportions and numbers of Siglec-F_int_ and Siglec-F^hi^ eosinophils, and **(F)** ST2^+^ ILC2s in the lungs at 5 days post-primary and secondary *Nb* infection. Data is representative of two independent experiments (5 mice per group). Statistical analysis was performed using a Mann Whitney t test or a two-way ANOVA with Bonferroni’s multiple comparisons tests. **p ≤ 0.01. #, Cell numbers.

We showed that Siglec-F^hi^ eosinophils expand in the lungs after parasite migration through the compartment (**Figure 1E**). In immunocompetent mice, *Nb* infection is naturally cleared after ~ 2 weeks (33). We observed that levels of Siglec-F^hi^CD101^hi^ eosinophils remained significantly elevated in the lungs at 6 weeks post *Nb* infection (**Figure 5A**). We also observed a trend for elevated eosinophil proportions in the bone marrow at this time point (**Figure 5B**), which is the probable source for recruited eosinophils in the lungs (34). Significantly increased numbers of lung ST2^+^ ILC2s at 6 weeks post *Nb* infection, compared to uninfected mice (**Figure 5C**), may contribute to the long-term maintenance of Siglec-F^hi^CD101^hi^ eosinophils in the lungs. Histological analysis of the lung tissue revealed that although *Nb* 6-week PI eosinophils retained Siglec-F^hi^CD101^hi^ expression, their morphology resembled that of steady-state lung eosinophils (**Figure 5D**). We also found significantly elevated numbers of Siglec-F^hi^ eosinophils and a trend for increased ST2^+^ ILC2s in the lungs during a recall infection, when compared to primary infection (**Figures 5E, F**). These findings demonstrate the long-term maintenance of Siglec-F^hi^CD101^hi^ eosinophils in the lungs long after parasite clearance, which is associated with lung ILC2s.

## Discussion

In this study, we have characterized the long-term induction of a Siglec-F^hi^CD101^hi^ eosinophil subpopulation in the lungs following *Nb* infection and identified that this expansion is strongly associated with the recruitment of lung ILC2s.

Previous studies have identified eosinophil subpopulations in mouse models of allergic lung inflammation (2, 3, 6). Percopo et al., 2017 identified a distinct subset of allergen-induced Siglec-F^+^Gr1^hi^ lung eosinophils that produce immunomodulatory cytokines (35), while Mesnil et al., 2016 differentiated homeostatic Siglec-F^int^CD62L^+^CD101_lo_ lung eosinophils and recruited pro-inflammatory Siglec-F^hi^CD62L^-^CD101^hi^ eosinophils following an allergic challenge (2). CD101-expressing eosinophils have also been identified in the blood and biopsies of allergic patients and are suggested to be pathogenic (4, 5). Our identification of *Nb*-induced Siglec-F^hi^ lung eosinophils expressing high levels of CD101 and presenting an activated morphology (hyper-segmented nuclei and dispersed ECP+ cytoplasm), compared to steady-state and *Nb*-expanded Siglec-F_int_ eosinophils, may indicate the accumulation of a pathogenic eosinophil subpopulation in the lungs following *Nb* infection.

Mesnil et al., 2016 demonstrated the induction of Siglec-F^hi^CD101^hi^ eosinophils during allergic lung inflammation to be dependent on IL-5 (2). Moreover, inflammatory Siglec-F^hi^CD101^hi^ eosinophils had increased the expression of type 2 receptor *Il13ra1* (2). We found the expansion of Siglec-F^hi^CD101^hi^ eosinophils in the lungs following *Nb* infection to be exclusively reduced in IL-4Rα^-/-^ mice, suggesting an additional requirement for IL-4 and/or IL-13 signaling.

Lung ILC2s are early responders to helminth migration through the lungs, producing IL-5 and IL-13 (23, 24, 36). Huang et al., 2018 showed intestinal ILC2s acquire an inflammatory phenotype (ST2^-^KLRG1^hi^) following helminth infection and migrate to peripheral sites, including the lungs (27). We did not observe this inflammatory ST2^-^KLRG1^hi^ ILC2 phenotype in BALB/c *Nb*-infected mice, which is consistent with the findings of ILC2 studies in BALB/c mice (28). However, we found ST2^+^(KLRG1^+/-^) ILC2 accumulation in the lungs peaked at day 9 post-infection, with increased levels of ILC2s in circulation at this time point. These findings support ILC2 accumulation in the lungs to be, to a large extent, a result of the recruitment from other sites such as the intestine (27). This lung ILC2 expansion mirrored the peak accumulation of Siglec-F^hi^CD101^hi^ eosinophils in the lungs. Through their production of type 2 cytokines, helminth-induced inflammatory ILC2s are known to regulate eosinophil accumulation (15, 37). Moreover, Huang et al., 2018 reported that inflammatory/recruited ILC2s produced more IL-13 compared to tissue-resident ILC2s (27). We found that expansion of Siglec-F^hi^CD101^hi^ lung eosinophils following hookworm infection was reduced following FTY720-mediated inhibition of ILC2 recruitment to the lungs. This finding identifies helminth-elicited ILC2s to be strongly associated with the expansion of Siglec-F^hi^CD101^hi^ eosinophils in the lungs, possibly through their production of type 2 cytokines IL-5 and IL-13.

IL-33-responsive CD4^+^ T cells are also a source of type 2 cytokines in the lungs and are important for memory responses to limit *Nb*-induced lung damage and worm burden during a secondary infection (16, 38). However, studies have shown that CD4^+^ T cells are not essential for the expansion of helminth-induced lung eosinophils (23, 39). Likewise, we found that type 2 responses emanating from T cells were not essential for the expansion of Siglec-F^hi^CD101^hi^ eosinophils in the lungs following *Nb* infection.

The distinct role of *Nb*-induced Siglec-F^hi^CD101^hi^ eosinophil subpopulations in the lungs is yet to be determined. Zhu et al., 2020 demonstrated that CD101^+^ eosinophils increase neutrophilic inflammation in the lungs, while CD101^-^ eosinophils reduced neutrophil inflammation (40). In the context of *Nb* infection, we found a reduction in Siglec-F^hi^CD101^hi^ eosinophils following FTY720 treatment to be associated with an increase in lung neutrophils; however, this could also be due to other effects such as impaired M2 macrophage activation in the lungs following FTY720 treatment (41).

We observed the long-term maintenance of *Nb-*induced ECP^+^ Siglec-F^hi^CD101^hi^ lung eosinophils at 6 weeks post-infection; however, their morphology resembled that of steady-state lung eosinophils. At this time point, mice typically develop emphysema-like pathology (13). Marsland et al., 2008 reported an equivalent long-term emphysema-like pathology in WT and IL-4Rα^-/-^ *Nb*-infected mice (13). Further investigation is needed to determine the role of *Nb*-induced, IL-4Rα-dependent Siglec-F^hi^CD101^hi^ lung eosinophils in long-term lung pathology.

We also observed an increase in lung Siglec-F^hi^ eosinophils following a recall challenge. It is unclear whether *Nb*-induced Siglec-F^hi^CD101^hi^ eosinophils have an enhanced ability to respond to secondary parasite infection or play a role in limiting tissue damage (16, 38). The long-term presence of *Nb*-induced Siglec-F^hi^CD101^hi^ eosinophils in the lungs may also have bystander effects on unrelated infections or allergic inflammation (15, 40, 42).

In summary, this study identifies that *Nb* infection drives the expansion of a morphologically distinct Siglec-F^hi^CD101^hi^ eosinophil subpopulation in the lungs. Helminth-induced ILC2 recruitment to the lungs contributes to Siglec-F^hi^CD101^hi^ eosinophil expansion. This eosinophil subpopulation persists after the resolution of parasite infection. This identifies these cells as an important associate with longer-term lung pathology following helminth migration through the lungs.

## Data availability statement

The original contributions presented in the study are included in the article/**Supplementary Material**. Further inquiries can be directed to the corresponding authors.

## Ethics statement

The animal study was reviewed and approved by University of Cape Town Faculty of Health Sciences Animal Ethics Commitee.

## Author contributions

AC, MD, AT and JP performed experiments, maintained the parasite lifecycle, and analyzed data. WH, AC, MR, LEL, GK, AC and MO designed experiments, prepared figures, and wrote the paper. WH, AFC, LEL, GK and MR acquired funding and supervised the work. All authors contributed to the article and approved the submitted version.
