# Supplementary material for: Induction of Siglec-FhiCD101hi eosinophils in the lungs following murine hookworm Nippostrongylus brasiliensis infection
Source: Front Immunol. 2023 May 12;14:1170807. doi: 10.3389/fimmu.2023.1170807 (PMC10213982; doi:10.3389/fimmu.2023.1170807)
Supplement: Supplementary file 1 [file DataSheet_1.pdf]

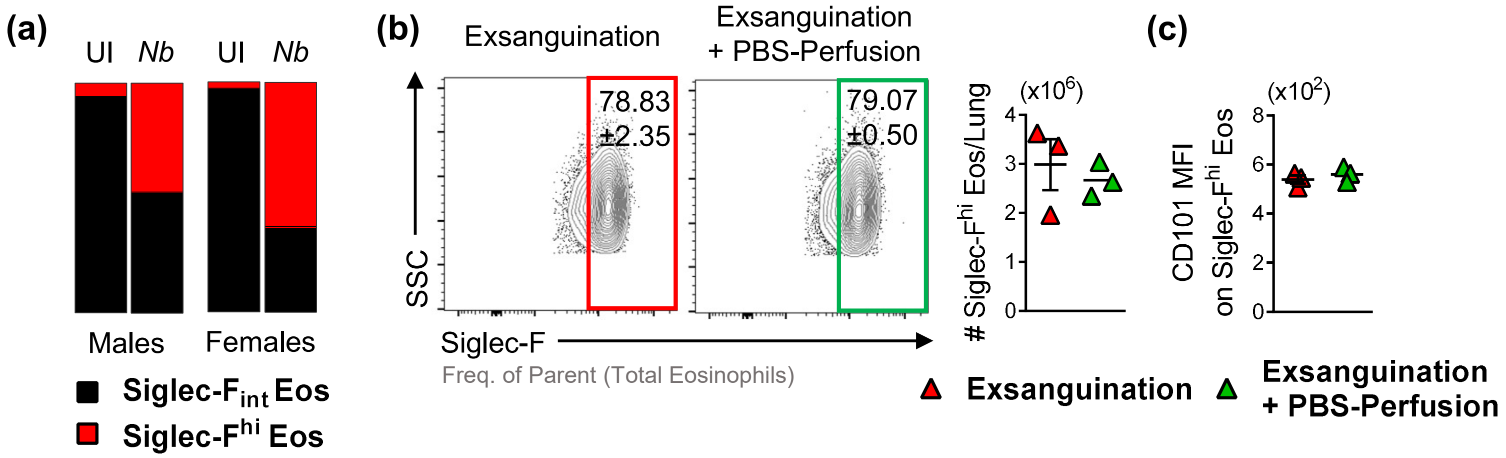

Supp Figure 1. Expansion of Siglec-F^{hi} lung eosinophils following *Nb* infection is independent of gender: (a) Mean proportions of Siglec-F^{hi} (red) and Siglec-F$_{int}$ (black) lung eosinophils in uninfected (UI) and *Nb* 9dpi male and female mice. Data is representative of two independent experiments (4-6 mice per group). **(b)** Proportions and numbers of Siglec-F^{hi} eosinophils and **(c)** mean CD101 expression on Siglec-F^{hi} eosinophils in the lungs following exsanguination (via cardiac puncture) and PBS-Perfusion. Data is representative of one experiment (3 mice per group).

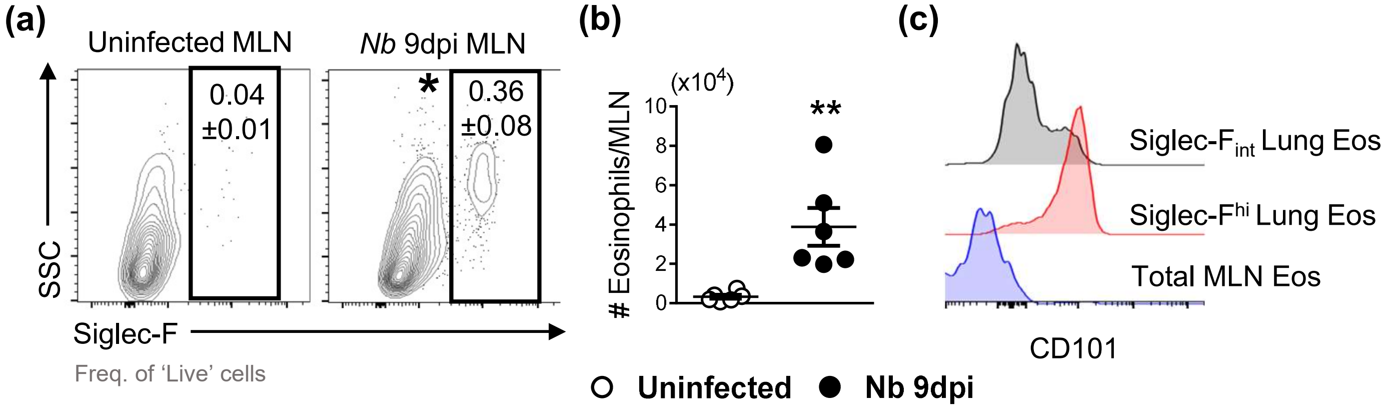

Supp Figure 2. *Nb* infection does not induce Siglec-F^{hi} eosinophils in the intestine-draining mesenteric lymph nodes (MLN): (a) Proportions and **(b)** numbers of total eosinophils in the MLN of uninfected and *Nb* 9dpi mice. **(c)** Representative histograms of CD101 expression on lung Siglec-F^{hi} (red) and Siglec-F$_{int}$ (black) eosinophils, and total MLN eosinophils (blue) at *Nb* 9dpi. Data is representative of two independent experiments (6 mice per group). Statistical analysis was performed using a Mann Whitney t test. **p* ≤ 0.05, ***p* ≤ 0.01.

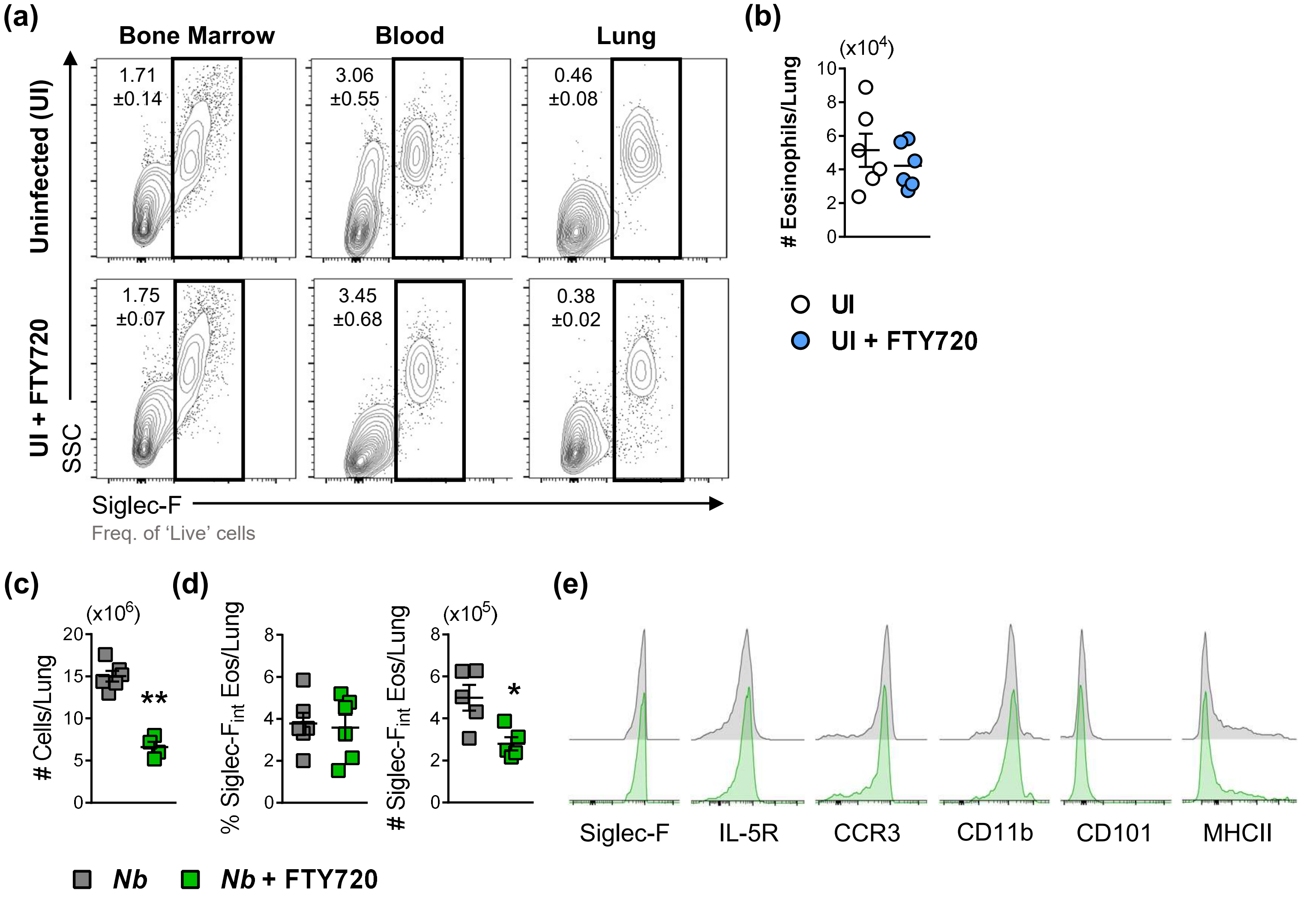

Supp Figure 3. FTY720 treatment does not significantly alter Siglec-F$_{int}$ eosinophil phenotypes in the lung: (a) Frequencies (% of 'Live' cells) of total eosinophils in the bone marrow, blood and lung at steady-state/uninfected (UI) and following FTY720 treatment (UI + FTY720). **(b)** Numbers of lung eosinophils in UI and UI + FTY720 mice. Data is representative of one experiment (6 mice per group). **(c)** Total lung cell numbers in WT untreated and FTY720-treated mice at *Nb* 9dpi. **(d)** Frequencies (% of 'Live' cells) and numbers of Siglec-F$_{int}$ lung eosinophils. **(e)** Representative histograms of Siglec-F, IL-5R, CCR3, CD11b, CD101 and MHCII expression on Siglec-F$_{int}$ lung eosinophils in untreated and FTY720-treated *Nb* 9dpi mice. Data is representative of two independent experiments (3-6 mice per group). Statistical analysis was performed using a Mann Whitney t test. *$p \leq 0.05$, **$p \leq 0.01$.
